# *Deja Tu Huella*: a comprehensive review of a successful emergency department-based, HIV-targeted screening program in Spain and its impact on early diagnosis and public health

**DOI:** 10.3389/fpubh.2025.1734172

**Published:** 2026-01-12

**Authors:** Juan González del Castillo, Domingo Mérida, Ivana Olla, Cristina De Álvaro, Jordi Llaneras Artigues, Manuel J. Vázquez Lima, Concepción Abellás, Oscar Miró, on behalf of SEMES Regional Coordinators

**Affiliations:** 1 Emergency Department, IdISSC, Complutense University, Madrid, Spain; 2 Gilead Sciences, Madrid, Spain; 3 Emergency Department, Hospital Universitari Vall d’Hebron. Barcelona, Barcelona, Spain; 4 Emergency Department, Hospital do Salnes, Pontevedra, Spain; 5 Pontevedra and do Salnes Health Area, Servicio Galego de Saúde, Pontevedra, Spain; 6 Emergency Department Hospital Clinic IDIBAPS, University of Barcelona, Barcelona, Spain

**Keywords:** early diagnosis, emergency department, HIV screening, implementation science, linkage to care, public health

## Abstract

Failure to diagnose human immunodeficiency virus (HIV) and late HIV diagnosis persist across Europe and Spain, sustained by missed diagnostic opportunities in routine care. Emergency departments (EDs), often the sole point of contact for vulnerable groups, are pivotal yet underused venues for early case finding. In response, the Spanish Society of Emergency Medicine (SEMES), with scientific sponsorship of the AIDS Study Group (GESIDA), published a consensus document recommending a targeted, opt-in HIV testing strategy in EDs triggered by specific HIV indicator conditions. This first consensus also emphasized standardized communication of results and linkage pathways to infectious disease care, and the use of electronic decision-support systems (preconfigured analytical panels and pop-up alerts) to normalize test ordering within ED workflows. An updated consensus later added further indicator conditions and recommended bundling hepatitis C virus (HCV) serology with every HIV test, supported by electronic health record (EHR)-based decision-support tools, clear linkage-to-care pathways and expanding the role of nursing. *Deja Tu Huella* translates these recommendations into routine ED practice at scale. Launched in 2021, the program combines multi-stakeholder governance, standardized clinical pathways, EHR-integrated order sets and alerts, and structured training for ED teams. By 2025, 187 hospital EDs adopted the program, requesting 223,659 HIV serologies and identifying 2,357 new cases (mean positivity: 1.15%). Participating EDs account accounted for >20% of national diagnoses, with rapid linkage to antiretroviral therapy (median: 4–9 days). The program has also generated a substantial body of peer-reviewed research that has iteratively informed updated SEMES recommendations. The initiative was co-developed by SEMES in collaboration with Gilead Sciences, which supported education, data generation, and deployment of EHR-based decision-support tools. Overall, *Deja Tu Huella* demonstrates that an indicator condition-based, opt-in HIV testing strategy, embedded in ED workflows and supported by decision-support tools and education, can achieve high diagnostic yield and timely linkage to care. This experience offers a scalable template for ED-based HIV and HCV screening that may be adaptable to other health systems seeking to accelerate progress toward the UNAIDS 95–95–95 targets.

## Introduction

Human immunodeficiency virus (HIV) remains a major global public health issue. Undiagnosed HIV is still a concern worldwide: in 2024, 5.3 million people were unaware that they were living with HIV (1). This is a particular issue in the European region, where nearly 113,000 diagnoses were reported in 2023, representing an increase of 2.4% compared to the previous year (2). In Spain, 3,350 new diagnoses were documented in 2023, most of which were attributed to sexual transmission. Late diagnosis remains a persistent challenge, affecting nearly half of newly diagnosed cases. This delay compromises clinical outcomes and perpetuates transmission, emphasizing the need for timely testing and intervention strategies (3–5). In this context, the Joint United Nations Program on HIV/AIDS (UNAIDS) has set their 2025 95–95–95 global target, that is to say 95% of all people living with HIV (PLWH) know their HIV status, 95% of all people diagnosed with HIV receive sustained antiretroviral therapy, and 95% of all people receiving antiretroviral therapy have viral suppression. This is complemented by their 10–10–10 societal enabler targets: less than 10% of countries should have punitive legal and policy environments that deny or limit access to services, less than 10% of PLWH and key populations will experience stigma and discrimination, and less than 10% of women, girls, PLWH, and key populations will experience gender inequality and violence. The goal is to reduce stigma, discrimination, gender-based inequalities and punitive legal barriers, and to keep countries on track to end AIDS as a public health threat by 2030 (6).

Late diagnosis is closely associated with a high rate of missed diagnostic opportunities (MDOs). Research across Europe has quantified the extent of MDOs, demonstrating that many diagnosed individuals had prior contact with medical professionals during which testing could have been offered. A study conducted at a Swiss university hospital found that 47% of newly diagnosed HIV patients had at least 1 missed opportunity for testing in the 5 years preceding their diagnosis (7). A multicenter study performed across several healthcare centers in Greece found an MDO rate of 52.3% among patients who had contact with the healthcare system before their HIV diagnosis. Recognizing these opportunities could have reduced the time to diagnosis by approximately 1 year (8). In Portugal, a cohort study revealed that 27.1% of individuals diagnosed with HIV had experienced at least 1 MDO in the 2 years prior to their diagnosis. These missed opportunities were significantly associated with a higher likelihood of having an acquired immunodeficiency syndrome (AIDS)-defining condition at the time of diagnosis (9). This is a common issue also reported in Spain. A study published in 2019 reported that over 86% of newly diagnosed individuals in the region of Aragón had at least 1 MDO in the previous 3 years (10). In line with these observations, a recent population-based study in Catalonia (2017–2021) found that across 372,712 primary care indicator episodes only 22.7% were followed by an HIV test within 4 months, a rate that was even lower among women and adults ≥50 years, clearly highlighting persistent MDOs in routine care (11).

Emergency departments (EDs) are critical yet often underutilized settings for diagnosing HIV. Patients with undiagnosed HIV frequently visit EDs with a range of clinical presentations or complaints that indicate a higher risk of HIV infection prior to diagnosis, but these visits often result in MDOs for earlier detection and timely intervention. As such, EDs can play a key role in addressing MDOs. A retrospective study in Southern Alberta, Canada, found that EDs accounted for 33.1% of all previous healthcare visits among patients with MDOs. The study highlighted cases where patients attended ED multiple times with HIV indicator conditions, such as lymphoma or pneumonia, but were not tested for HIV (12). Similarly, a study in New York City identified EDs as the most frequent sites for MDOs, accounting for 48.7% of cases (13). In Spain, nearly 1/3 of MDOs are traced to EDs (10, 14), while patients who have prior ED visits have a significantly higher risk of receiving a late HIV diagnosis (15). A study in Aragón found that the rate of late HIV diagnosis increased with the number of previous ED visits: 35.6% with no visits, 51.8% with 1 visit, and 58.1% with multiple visits. On average, newly diagnosed individuals had 2.1 ED visits in the preceding 3 years (10). A Spanish multicenter study found that 16.3% of PLWH had visited an ED without being diagnosed, despite presenting with suggestive symptoms (14). Similarly, a single-center study showed that over 55% of individuals tested for sexually transmitted infections (STIs), including chlamydia or gonorrhea, were not simultaneously tested for HIV, despite a high prevalence of HIV in this population (1.7%) (15). Moreover, EDs frequently serve as a common or, sometimes, the only point of contact with the health system for vulnerable groups (explicitly so in the case of people experiencing homelessness and frequently for international migrants, due to barriers to receiving timely primary care). Accordingly, provider-initiated HIV testing within secondary-care pathways can help reach migrants who disproportionately rely on EDs (16, 17). Likewise, people experiencing homelessness commonly use EDs as their principal source of healthcare, underscoring the value of ED-embedded HIV screening to reduce MDOs and late presentations (18). Taken together, these results highlight EDs as an underutilized opportunity for early HIV diagnosis and intervention. They are not only a environment with a high risk of late presentations, but also feature broader systemic shortcomings: 2/3 of Spanish EDs lack specific protocols for managing suspected HIV cases, contributing to delayed diagnosis and care (10, 14, 19).

In response to this need, several countries have implemented ED-based HIV screening strategies. In England, routine opt-out HIV testing, where patients are systematically offered an HIV test unless they explicitly decline, was introduced in high-prevalence areas in April 2022, with the goal of integrating HIV screening into standard emergency care practices (20, 21). A key strength of this approach lies in its seamless integration into existing ED workflows, often facilitated by information technology (IT) that automates test requests and minimizes disruptions to patient flow. In France, 2 notable implementation strategies in Paris illustrate the diversity and effectiveness of ED-based screening models. One initiative conducted across 6 EDs used an opt-in model without dedicated staff, achieving a 3.9% screening rate and a 0.61% diagnosis rate (22). Additionally, a cluster-randomized trial showed that combining nurse-led targeted screening with physician-directed testing increased new HIV diagnoses from 0.008 to 0.03% among included ED patients, of whom 92.9% completed a risk-assessment questionnaire (23).

Given the pivotal role of EDs in addressing missed HIV diagnoses, the *Deja Tu Huella* initiative was launched in Spain in 2021. This opt-in program aims to achieve a meaningful and sustained improvement in the differential diagnosis of HIV in EDs by integrating decision-support tools that assist clinicians in ordering HIV serology. The initiative also serves as a platform for delivering educational activities targeting both healthcare professionals and patients, while fostering ongoing collaboration among participating hospitals (24).

This review aims to examine the development, implementation and impact of the *Deja Tu Huella* initiative and explore its potential as a model of HIV diagnosis and care for broader adoption across other healthcare systems.

## Methods

This article is a narrative review of the design, implementation and outcomes of the *Deja Tu Huella* program. We synthesized information from three main sources: (i) peer-reviewed publications and conference abstracts arising from *Deja Tu Huella* and closely related ED HIV screening work; (ii) internal SEMES *Deja Tu Huella* program documents (including the 2021–2023 Deja Tu Huella Memoir) and routine aggregated monitoring reports; and (iii) independent external literature on HIV epidemiology and ED-based testing strategies in Spain and comparable settings. A list of *Deja Tu Huella*–related primary publications is provided in Supplementary Table S1. No formal systematic review or meta-analysis was undertaken.

### *Deja Tu Huella*: origins and foundations of the program

#### Before the program

The initial recommendations issued by the Spanish Ministry of Health in 2014 proposed a comprehensive, three-tiered strategy for HIV testing within healthcare settings. This framework included: (i) mandatory testing for individuals presenting with clinical symptoms or conditions suggestive of HIV or AIDS; (ii) a “directed offer” of testing for asymptomatic individuals at increased risk, including men who have sex with men, people who inject drugs, sex workers, and individuals from high-prevalence countries; and (iii) the normalization of screening through a “routine offer” to the general sexually active population (ages 20–59) in high-incidence areas, pregnant women, and incarcerated individuals. Additionally, mandatory testing was required for all blood and organ donations (25).

However, despite the comprehensive nature of these guidelines, they were ultimately considered too generic and were not effectively implemented in clinical practice. For instance, a national survey conducted among Spanish EDs revealed that 65.2% of ED physicians reported “almost never” or “infrequently” ordering HIV serology, even in the presence of a condition that should have led to HIV screening (26). Another study published in 2021 found that only 36% of departments had a specific protocol for STIs, 70% performed specimen collection for exudates, 44% requested STI serologies, 35% requested HIV serologies, 53% referred patients for follow-up to hospital services, and 28% to primary care. Notably, only 55% of departments reported frequently or almost always scheduling a follow-up visit (19).

#### SEMES recommendations for EDs

In response to the scenario described above, the Spanish Society of Emergency Medicine (SEMES), with the scientific sponsorship of the AIDS Study Group (GESIDA), published a consensus document with recommendations on HIV screening in EDs (27). This document reflects the effort to bring a tangible and lasting change to HIV diagnosis in routine clinical practice, while serving as a model of best practice aimed at achieving the UNAIDS 95–95-95 targets. Based on considerations of efficiency, clinical relevance, and organizational feasibility, this document does not recommend screening every patient who visits an ED, but rather strategically targeting individuals who present specific clinical profiles with a higher likelihood of undiagnosed HIV infection: in other words, an opt-in HIV diagnostic approach. Compared to universal screening, a focused program targeting high-risk populations is more feasible and sustainable, as it involves a significantly lower testing volume (27). In line with European public health guidance, we refer to these clinical presentations as HIV indicator conditions and to the associated approach as a targeted, opt-in testing strategy, whereby HIV serology is routinely offered to eligible patients but performed only with explicit patient consent.

SEMES identified 6 clinical indicator conditions to trigger this opt-in HIV testing strategy: STIs; community-acquired pneumonia (CAP); post-exposure prophylaxis (PEP); herpes zoster (HZ); mononucleosis-like syndrome (MNS); and chemsex practices. These indicator conditions were selected by an expert panel convened by SEMES, comprising specialists in Emergency Medicine, Infectious Diseases, and Microbiology on the basis of 2 main criteria: (i) an increased prevalence of HIV (with respect to the general population) or a high risk of acquisition among patients with these indicator conditions; and (ii) their frequent appearance among acute consultations or diagnoses in EDs (27). These indicator conditions are indeed common in emergency care: 75% of CAP cases (28) and 80% of MNS consultations (29) are managed directly in hospital EDs. This convergence of high HIV association and high ED throughput supports the use of these indicator conditions as focal points for targeted screening to improve early diagnosis. Although other conditions are also linked to HIV, SEMES limited the initial recommendations to 6 indicator conditions to promote feasible, sustainable implementation. The guidance further emphasizes that testing must remain voluntary and confidential and that explicit oral consent must be sought from patients after informing them of the procedure’s risks and benefits (27).

### *Deja Tu Huella*: an efficient way to implement SEMES recommendations

#### Structure: a collaborative public health program

The incorporation of a differential HIV diagnosis into routine practice in hospital EDs has been the cornerstone of an ambitious scientific initiative led by SEMES. Acknowledging the complexity of changing entrenched clinical behaviors, SEMES adopted a forward-thinking approach aimed at translating medical-expert consensus into operational protocols.

To realize this vision, SEMES co-created and jointly implemented the *Deja Tu Huella* program in collaboration with Gilead Sciences. This innovative public health initiative is grounded in multi-stakeholder collaboration and designed to promote the adoption of SEMES recommendations while strengthening referral pathways for patient follow-up^1^.

The *Deja Tu Huella* program gave rise to the *Deja Tu Huella*-HIV network, an integrated, collaborative framework that brings together key stakeholders across the healthcare ecosystem. The network is structured around a 4-pillar model to maximize its effectiveness and scalability (24):

- SEMES acts as the central coordinating entity, managing a multi-tiered structure with clearly defined roles across various levels of the healthcare system.
- Scientific and civil society organizations form the second pillar, with societal guidance from CESIDA (Spanish State Coordinator for HIV and AIDS, the leading umbrella organization representing civil society groups involved in HIV/AIDS advocacy in Spain), SEIMC (Spanish Society of Infectious Diseases and Clinical Microbiology), and its dedicated AIDS Study Group, GESIDA.
- Health authorities and ED leadership comprise the third pillar, providing institutional support and enabling integration within the broader public health infrastructure.
- Gilead Sciences, a pharmaceutical company, provides substantial support for the development and implementation of the program. This includes funding for educational initiatives, data generation, and the deployment of electronic tools to facilitate clinical decision-making.

**Figure 1:**
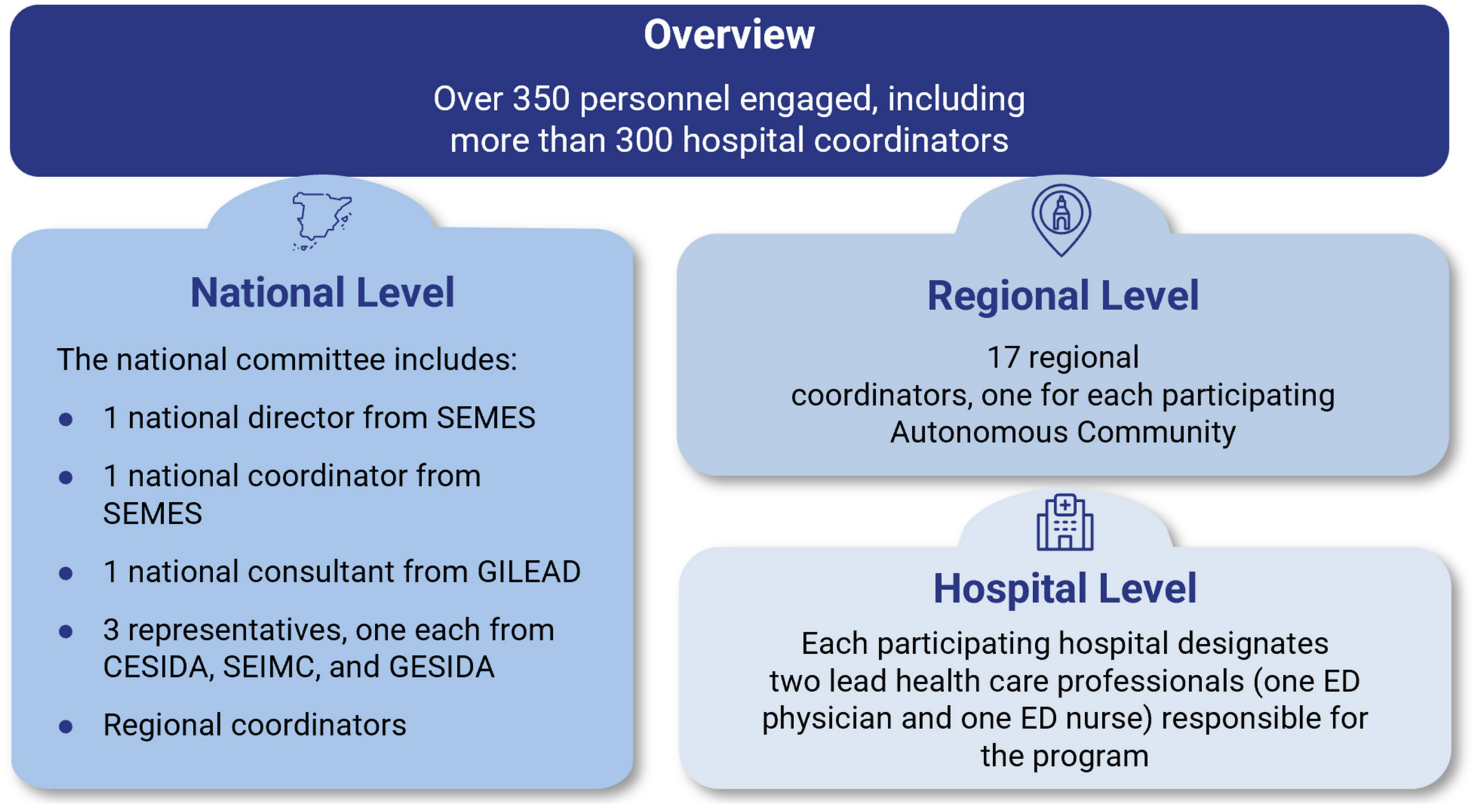
*Deja Tu Huella* multilevel organization structure. ED, emergency department; CESIDA, Spanish State Coordinator for HIV and AIDS; GESIDA, AIDS Study Group; SEIMC, Spanish Society of Infectious Diseases and Clinical Microbiology; SEMES, Spanish Society of Emergency Medicine.

Currently, the *Deja Tu Huella*-HIV network involves 187 hospital EDs, with over 350 healthcare professionals, spanning 16 of Spain’s 17 autonomous communities and including tertiary referral centers, general hospitals and smaller community/county hospitals. This supposes around 65% of all Spanish hospital EDs operating 24/7 of the whole national public health network. In population terms, the program covers approximately 82.2% of Spain’s population (39.99 million of 48.62 million in 2024). Participating sites include the EDs of most large public hospitals and a mix of urban and rural services, together accounting for a substantial proportion of annual ED attendances nationally. As such, the network is broadly representative of the main settings in which acute care is delivered in Spain. Those local, regional, and national levels operate through a coordinated structure that includes local leads and a multi-organization national committee (Figure 1).

Each participating hospital designates an ED professional as a project manager who is responsible for coordinating local implementation and follow-up. Collectively, these project managers constitute a working group with 2 main objectives: (i) to maintain an open channel of collaboration among hospitals, particularly for program maintenance, educational initiatives, and research strategies; and (ii) to provide a platform for disseminating the outputs and as a point of contact for onboarding new sites (30). In Catalonia, this framework operates as *Urgències VIHgila* (https://www.urgencies-vihgila.cat) and in Galicia as *Urgencias VIHxia* (https://www.sergas.es/Saude-publica/URXENCIAS-VIHXIA). These regional adaptations of the national *Deja Tu Huella* initiative align local activities with national standards while accommodating regional conditions.

Ultimately, the *Deja Tu Huella* network aims to ensure adherence to the consensus document across all participating ED departments, on the understanding that deviations from guideline- or consensus-based recommendations in routine clinical practice are often substantial.

#### Methodology for implementation and sustainability

**Figure 2:**
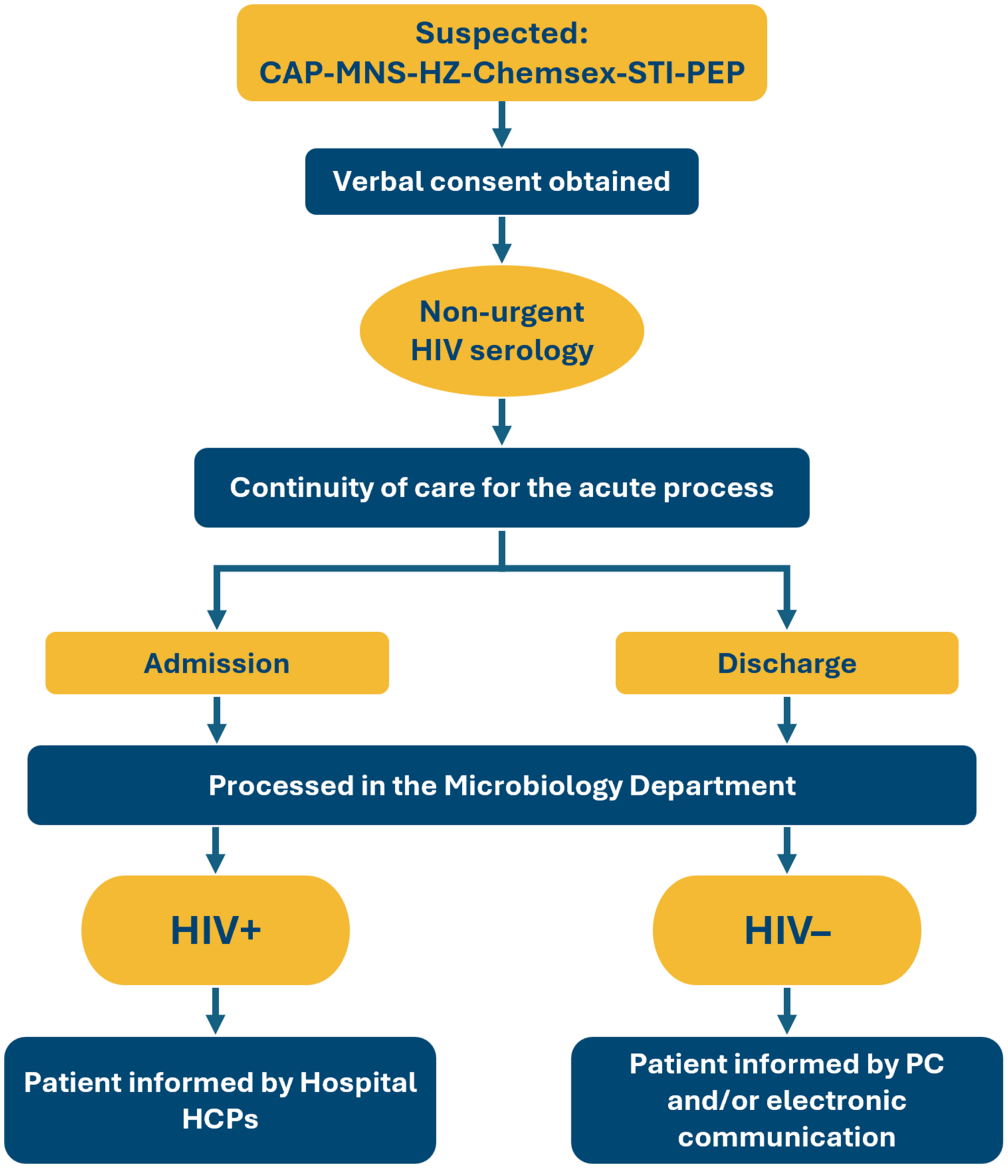
Test and linkage-to-care algorithm for hospital emergency departments proposed by the *Deja Tu Huella* program. Hospital health care professionals are primarily Infectious Diseases physicians or specialists in internal medicine/emergency department. CAP, community-acquired pneumonia; HCP, health care professional; HIV, human immunodeficiency virus; HZ, herpes zoster; ID, infectious diseases; MNS, mononucleosis-like syndrome; PC, primary care; PEP, post-exposure prophylaxis; STI, sexually transmitted infection.

*Deja Tu Huella* outlines a 3-pronged care algorithm, involving (i) the hospital ED, responsible for ordering HIV serology; (ii) the microbiology department, which performs the serologies and initiates referrals; and (iii) the infectious diseases department, which communicates results to patients and manages clinical follow-up (Figure 2). Each of the 187 hospitals that have implemented *Deja Tu Huella* adapts this algorithm locally, allowing for local flexibility while maintaining consistent compliance with the structured *Deja Tu Huella*-HIV network.

The initiative incorporates comprehensive communication and training strategies to address key barriers to HIV testing in EDs, the most notable being the perception among ED physicians that testing is not immediately necessary for clinical decision-making. Educational efforts emphasize the importance of early HIV diagnosis and its impact on patient prognosis, prevention of onward transmission, and reduction of healthcare costs (31, 32). Training was a core pillar of *Deja Tu Huella*. SEMES developed a standardized package of educational materials (presentations, case-based discussions and quick-reference tools) that local leads could adapt. No centralized, progra-wide evaluation of training has been performed, although local audits suggest that targeted education was associated with increases in HIV test ordering.

The *Deja Tu Huella* website (see footnote 1), which attracts approximately 36,000 unique users annually, offers a wide range of resources, including clinical tools and training materials designed to support autonomous implementation by participating hospitals. Dissemination is reinforced through regular updates via social media and newsletters, ensuring that healthcare professionals remain informed about emerging evidence and evolving strategies. Patient-facing materials are also placed in ED waiting areas to promote awareness and engagement (24).

Moreover, *Deja Tu Huella* proposes 2 groups of standardized, consensus-driven tools to support ED professionals ordering HIV serologies. These tools are designed to be user-friendly and seamlessly integrated into clinical workflows, ensuring that testing is considered whenever relevant clinical scenarios arise. By replacing manual processes with easily accessible buttons embedded into hospital IT systems, they streamline and enhance clinical decision-making. Nonetheless, the final decision remains at the discretion of the attending physician (27).

The first category includes preconfigured requests for profile-analytical laboratory panels embedded in the hospital’s electronic health record (EHR). These panels incorporate HIV serology when certain clinical conditions are selected and link automatically to each of the clinical indicator conditions included in the *Deja Tu Huella* program. For each clinical entity, a specific set of microbiological determinations is proposed, minimizing inter-individual variability in sample collection and test ordering practices (33).

The second category comprises pop-up alerts generated by specific algorithms implemented in the EHR when a patient consults the ED with a clinical scenario consistent with HIV infection. The primary objective is to identify potential candidates for serology based on a standardized set of criteria, ensuring that patients who meet specific risk profiles are flagged. Critically, in high-throughput EDs that are often saturated and weighed down by heavy clinical workloads, automatic flagging operationalizes standardized testing criteria at predictable workflow points, reducing omissions and bias and ensuring linkage to care even when clinicians are overstretched. Additional advantages include: (i) easy expansion of the testing criteria to encompass most of situations recommended by the Ministry of Health, beyond those originally outlined in the program; (ii) identification of patients that have recently received care for HIV-related conditions but have not yet undergone the corresponding serologies, enabling “rescue” testing in the ED regardless of the reason for the visit; (iii) identification of patients already diagnosed with HIV who are not receiving specialist follow-up or treatment, providing another opportunity to link them to care; and (iv) integration of messaging in the medical record stating that the patient has been informed whether the strategy is opt-in or opt-out and that consent for serology has been obtained. By confirming the message, the healthcare provider specifies in the medical record that the patient was informed, consent was requested, and testing was declined, thus preventing omissions (33).

Although specific technical implementations vary across hospitals and EHR vendors, *Deja Tu Huella* alerts are based on a shared, clinically driven logic. Alerts are triggered by predefined combinations of structured EHR data, including: prior HIV indicator conditions without documented testing, country of birth in high HIV-prevalence settings without prior diagnosis, reasons for consultation recorded at triage, selected diagnostic tests or treatments ordered in the ED, and diagnoses established during the ED encounter. This approach enables identification of both current and prior missed opportunities for HIV testing, including “rescue testing” scenarios.

To accommodate EHR heterogeneity, implementation was adapted locally, prioritizing structured data fields consistently available within each system. Where technically feasible, real-time pop-up alerts were integrated at predefined workflow points (e.g., order entry or diagnostic coding); in more constrained environments, preconfigured analytical order sets linked to indicator conditions served as the main decision-support tool.

To mitigate alert fatigue and support clinician acceptance, alerts were restricted to high-yield scenarios, embedded at predictable workflow moments, designed to avoid repeated firing within the same care episode, and allowed clinicians to document acceptance or refusal of testing. Final clinical decisions always remained at the discretion of the treating physician.

Although no formal, program-wide evaluation of usability has been conducted, sustained use over time, progressive expansion across hospitals, and greater increases in HIV testing in centers combining automated alerts with education support the feasibility and adoption of this approach.

Finally, to support continuous improvement and robust project oversight, the program conducts regular coordination meetings with stakeholders. This structured and proactive communication framework underpins the scalability and sustainability of *Deja Tu Huella*. Equally important is the systematic dissemination of results at the hospital (local), autonomous community (regional), and national levels, ensuring that clinicians receive timely feedback and can see that their day-to-day efforts yield tangible outcomes. Transparent reporting and feedback loops foster engagement, inform practice adjustments, and strengthen the program’s credibility across sites.

### New SEMES recommendations on best practices for *Deja Tu Huella*

Following the first 3-year evaluation of the *Deja Tu Huella* program, new recommendations have been established to strengthen and optimize HIV screening initiatives. These guidelines were developed through a formal, structured consensus process led by a designated SEMES group of experts in Emergency Medicine, Infectious Diseases, and Microbiology, based on scientific evidence accumulated throughout the project (33). The initial focus on 6 indicator conditions was the catalysis for a cultural shift in how ED professionals approach HIV diagnosis. By year-end 2023, the *Deja Tu Huella* initiative was active in 132 hospitals across 16 of Spain’s 17 autonomous communities. Participating EDs conducted nearly 130,000 HIV serologic tests and identified 1,620 previously undiagnosed infections, yielding an overall positivity rate of 1.2%. Test volume and case detection increased steadily over 2021–2023 (27). Once that shift had been consolidated across EDs, SEMES expanded the consensus to include new indicator conditions for HIV testing, namely fever of unknown origin, unexplained thrombocytopenia, and individuals from countries with high HIV prevalence, thereby increasing diagnostic opportunities (33). These three additions were selected for the following reasons: (i) the *Urgències VIHgila* evaluation across 20 EDs showed that these indicator conditions have acceptable diagnostic efficiency (34); (ii) they align with Spain’s Ministry of Health guidance, which explicitly lists fever of unknown origin and thrombocytopenia as HIV indicator conditions and supports testing for individuals from countries with HIV prevalence >1% (25); and (iii) they are frequent, well-defined ED presentations, making them suitable for standardized implementation and generalization (33).

Another significant advance has been the synergistic integration of hepatitis C virus (HCV) screening, which according to the new SEMES consensus should now be performed concurrently with every HIV serology (33). ED studies show an active HCV prevalence of ~0.35–0.70% among adults undergoing routine blood testing. This is higher than recent estimates in the general population (0.22%). Furthermore, ~51% of viremic patients present advanced fibrosis, and ~60% of viremic cases do not meet Spain’s national criteria for HCV serology, and thus are likely to be missed by selective strategies (35, 36). These data, together with the well-established overlap between injection-related and sexual transmission networks for HIV and HCV, justify bundling HCV and HIV serology in the same ED encounter, a strategy that aligns with the World Health Organization 2030 viral hepatitis elimination targets (37, 38). Furthermore, as with HIV, EDs interventions provide an opportunity to (re)link to care patients previously diagnosed with HCV who have not yet received the appropriate antiviral therapy. In practice, the *Deja Tu Huella* program embeds automatic flagging systems in routine ED workflows consisting of standardized preconfigured order sets and EHR alerts, so that HCV serology is automatically linked whenever HIV serology is requested, while minimizing duplicate or unnecessary testing (33).

Central to this expanded approach are robust protocols for patient consent and communication that emphasize informed, opt-in consent and establish clear procedures for result delivery and linkage to care (33). As already recommended in the 2020 SEMES consensus document (27), the use of technology to support implementation, such as EHR pop-up alerts and preconfigured analytical profiles, is essential for streamlining test requests and reducing missed diagnoses (33).

Moreover, expanding the role of nursing staff, particularly offering them targeted training and making them instrumental in obtaining consent to initiate serological testing, has emerged as a key lever for increasing testing rates. The involvement of nursing staff in HIV diagnosis within EDs in Catalonia was associated with an increase of more than 20% in HIV test requests (39). Consistent with these findings, the *Deja Tu Huella* program subsequently recommended the involvement of at least one nurse coordinator in each participating hospital, as reflected in the new consensus document (33).

Taken together, these updated recommendations reinforce the role of *Deja Tu Huella* as a dynamic, evidence-based program that can evolve in response to real-world practice, thereby ensuring its long-term sustainability and broader applicability.

### Impact and effectiveness of *Deja Tu Huella*

#### Scientific contributions

*Deja Tu Huella* generated several different publications between 2021 and 2025, including peer-reviewed original research (multicenter observational surveys and service mapping, a systematic review with meta-analysis, health-economic evaluations, before/after implementation studies; program evaluations of indicator condition testing, workforce training evaluations, and descriptive characterizations of ED HIV diagnoses); practice-oriented outputs (a national recommendation statement and a pragmatic implementation running order); scholarly communications (correspondence/brief reports); and conference abstracts/posters. In total, the program produced 21 original articles. Table S1 includes the peer-reviewed publications that present primary *Deja Tu Huella* data and constitute the main quantitative evidence base for this review. Internal SEMES *Deja Tu Huella* reports are used only to describe program implementation and reach, while external studies are cited to provide additional epidemiological and public health context.

#### Adherence to SEMES recommendations

A longitudinal study evaluated the impact of an HIV screening intervention across 34 EDs, comparing data from before (second semester of 2019) and after (first semester of 2022) implementation of the *Deja Tu Huella* program (40). The intervention combined an intensive educational program with the development of clinical pathways to facilitate and monitor HIV test ordering and outcomes. Adherence to the 6 SEMES HIV testing recommendations improved significantly across all indicator conditions except chemsex, where adherence was already high and remained stable. Post-implementation adherence rose notably for PEP (from 68.2 to 82.5%), STIs (from 36.2 to 66.8%), and MNS (from 26.5 to 51.1%), with more modest gains for CAP and HZ. The HIV test positivity rate increased from 0.92 to 1.67%. In addition, all 6 conditions showed a prevalence above the 0.1% cost-effectiveness threshold (41, 42). This threshold is derived from modeling studies in high-income settings, which indicate that routine HIV screening remains cost-effective when the undiagnosed HIV prevalence among those tested is ≥0.1%, assuming contemporary antiretroviral therapy costs and life expectancy (41, 42). Despite these encouraging results, significant gaps remained: approximately 80% of patients with CAP and over 70% of those with HZ were not tested in EDs where *Deja Tu Huella* had been implemented.

#### Implementation strategies

The implementation of the *Deja Tu Huella* strategies (27, 33) has produced a positive, measurable impact on HIV testing rates in hospitals that adopted them. A recent multicenter evaluation conducted in 2024—4 years after the launch of the program—compared hospitals implementing the bundle (educational program, preconfigured electronic order sets, and automated electronic alerts) with those without implementation and found statistically significant increases in overall testing, especially among patients with the 6 initially indicator conditions (STI, MNS, CAP, HZ, PEP, and chemsex). Greatest gains were found where automated alerts complemented education and preconfigured order sets. The improvement also extended, albeit more modestly, to patients without the 6 indicator conditions, in line with system-level facilitation effects (43).

**Figure 3:**
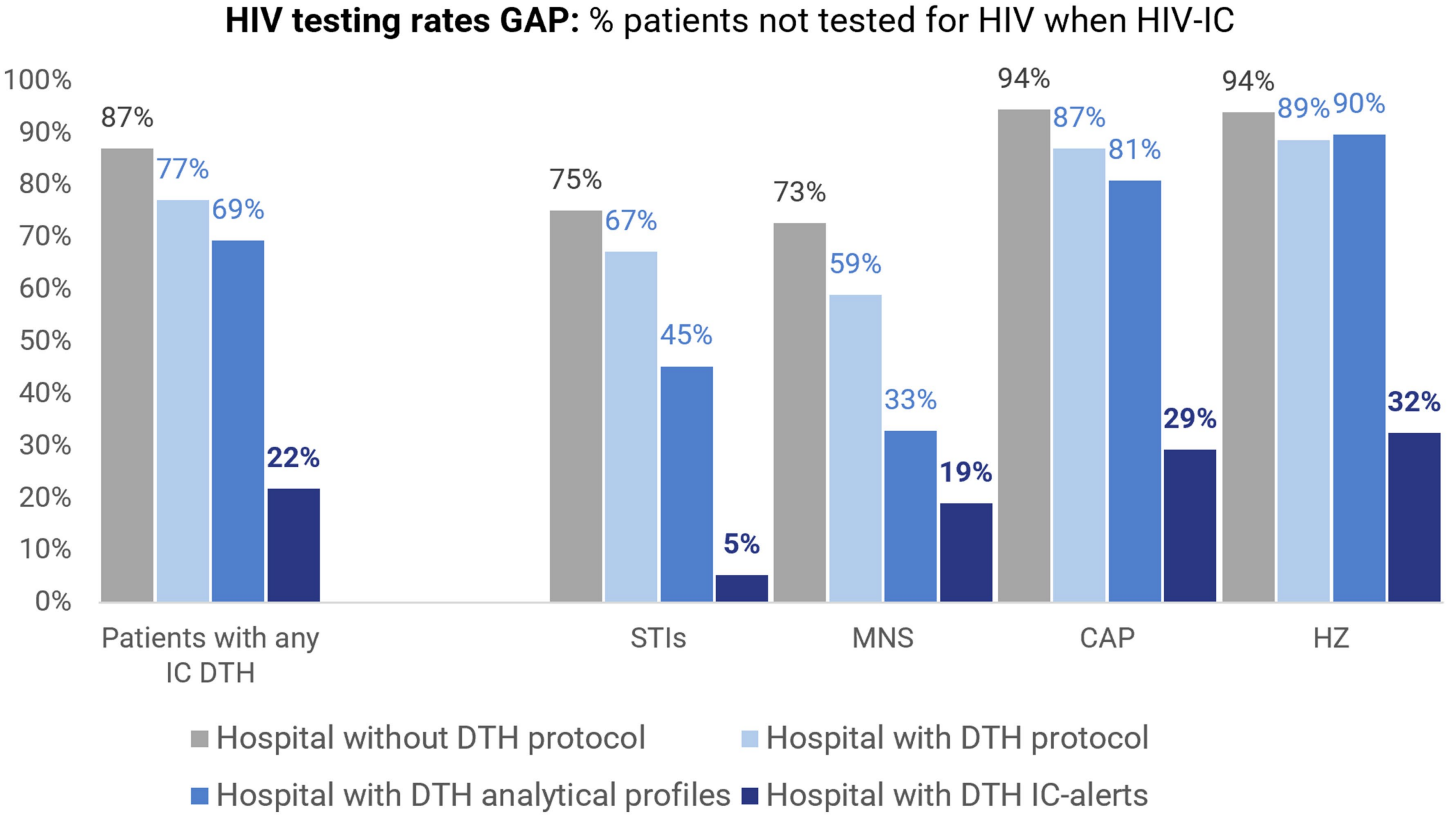
Proportion of patients in Spanish hospital emergency departments who were not tested for HIV, shown by implementation scenario and by indicator condition (IC). Bars display the aggregate of patients presenting with any of the first ICs prioritized in the *Deja Tu Huella* program (DTH) (27) and, separately, 4 ICs common in EDs and included in the DTH: sexually transmitted infections (STIs), mononucleosis-like syndrome (MNS), community-acquired pneumonia (CAP), and herpes zoster (HZ). Implementation scenarios: without protocol; DTH protocol; DTH protocol + DTH analytical profiles; and DTH protocol + DTH analytical profiles + DTH electronic IC-alerts. Adapted from González del Castillo et al. (43).

**Figure 4:**
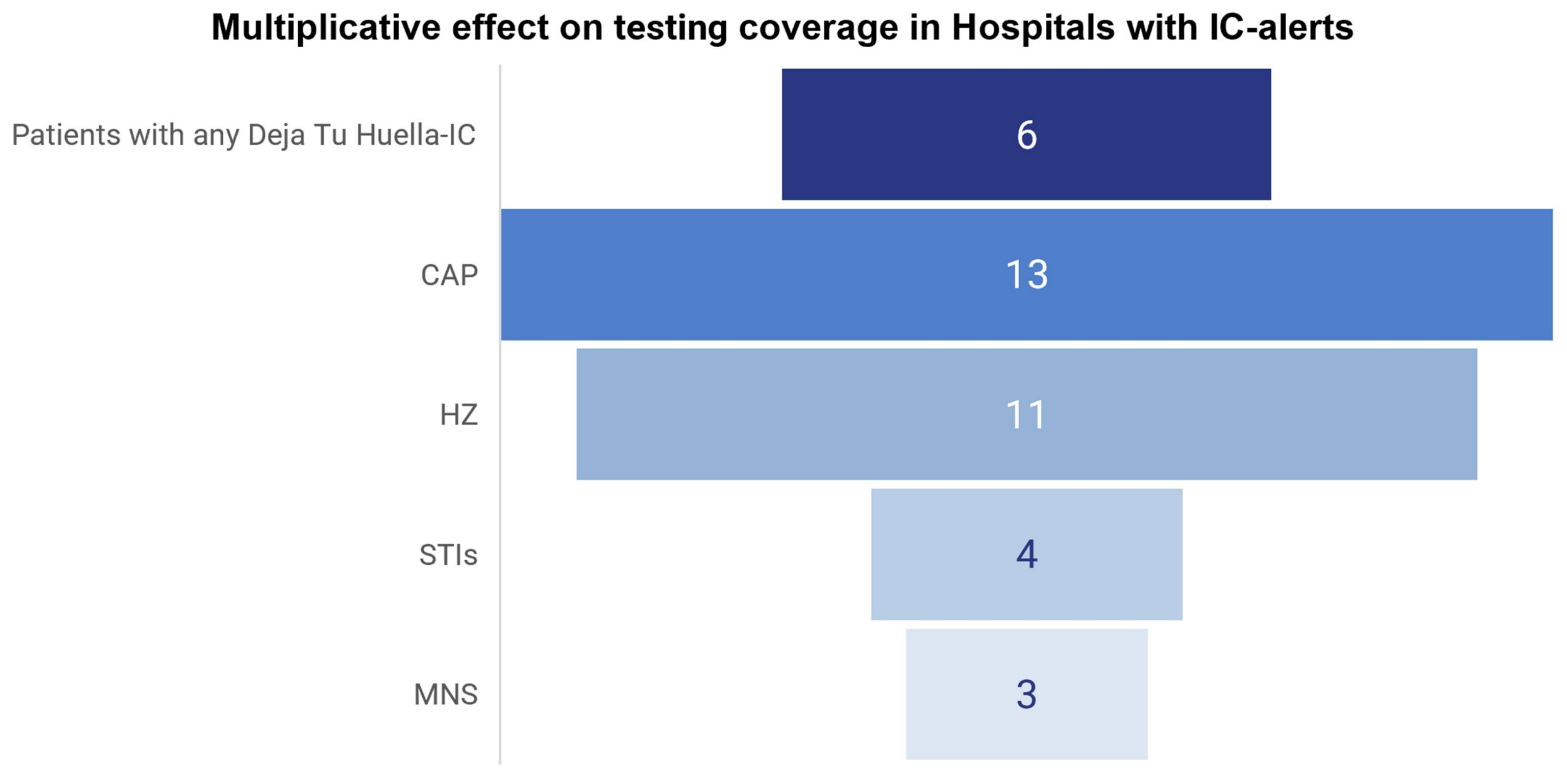
Multiplicative effect of indicator condition (IC) alerts on HIV testing coverage in Spanish hospital emergency departments. Bars show the fold-increase in the proportion of eligible patients tested for HIV in hospitals with IC alerts versus those without alerts, overall (patients with any of the first 6 ICs prioritized in the *Deja Tu Huella* program) (27) and by specific ICs: sexually transmitted infections (STIs), mononucleosis-like syndrome (MNS), community-acquired pneumonia (CAP), and herpes zoster (HZ). Fold-increase values were calculated using the data published in González del Castillo et al. (43).

When contrasted with the 2022 before/after analysis (40), the proportion of HIV patients tested rose across all 6 indicator conditions, with particularly notable gains for HZ, CAP, MNS and STIs, indicating consolidation of the impact over time. Consequently, the testing gap among patients with the prioritized conditions—especially HZ, CAP, MNS and STIs—has narrowed substantially (Figure 3), demonstrating the multiplicative effect of the *Deja Tu Huella* strategies on HIV testing rates among patients with the indicator conditions, likely driven by sustained implementation (Figure 4).

#### Outcomes

**Figure 5:**
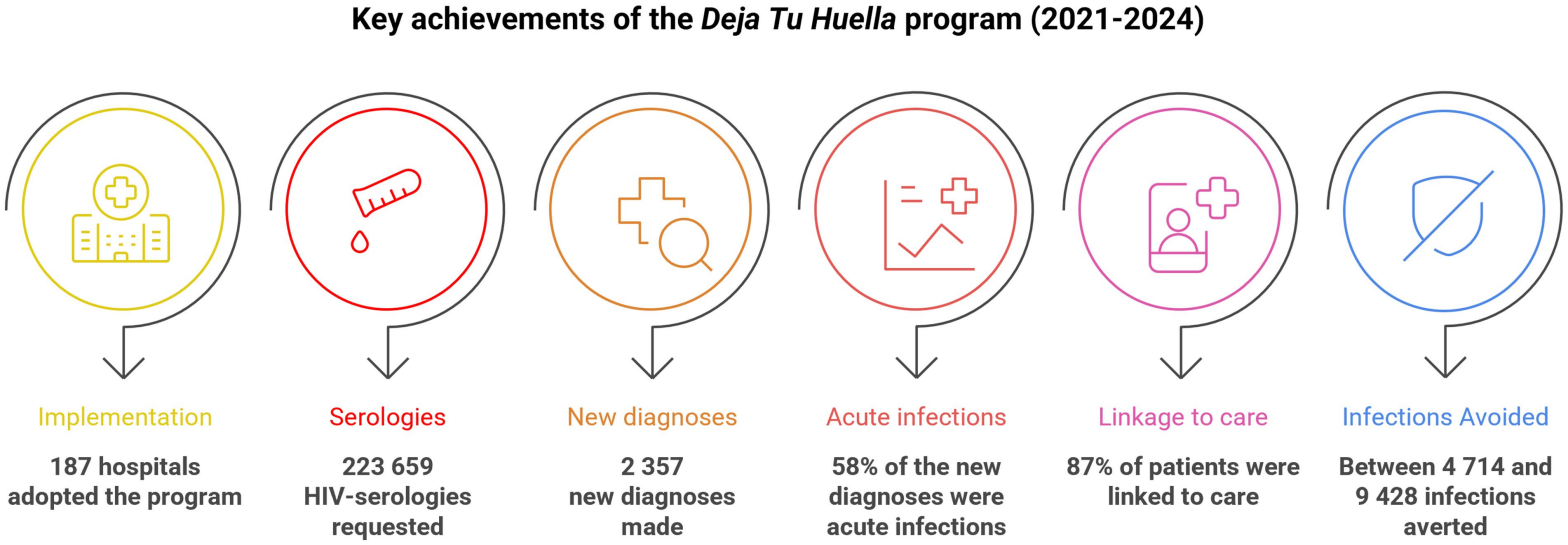
Key achievements of the *Deja Tu Huella* program (corresponding to the 2021–2024 period).

**Figure 6:**
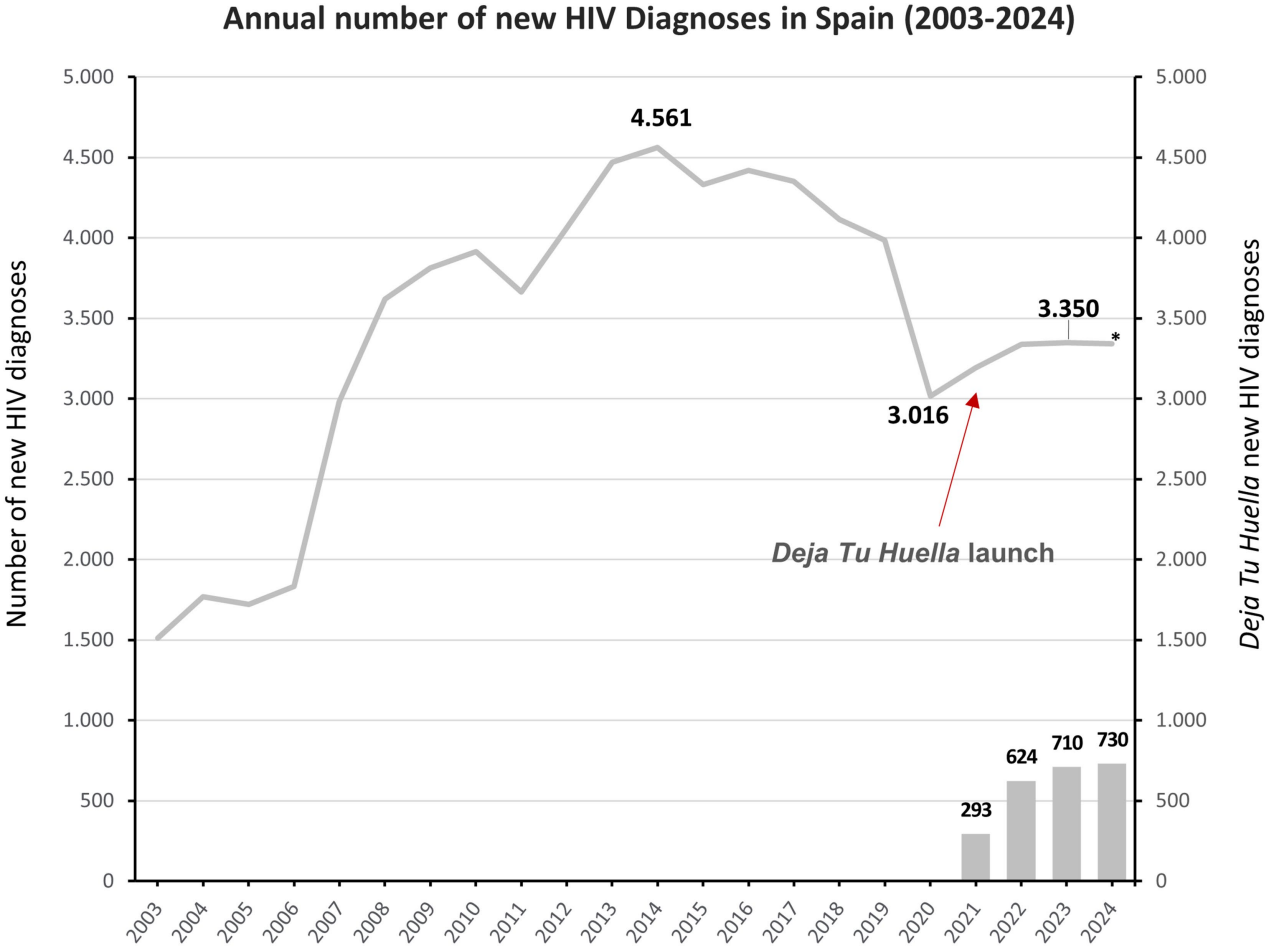
Annual new HIV diagnoses in Spain (2003–2024). The solid line shows national annual counts of newly reported HIV diagnoses (2003–2024). Bars depict diagnoses identified through the *Deja Tu Huella* program (2021–2024). A sharp decline is seen in 2020, coinciding with the onset of the COVID-19 pandemic. A change in the national trend is apparent after the 2021 launch of *Deja Tu Huella*. National pre-exposure prophylaxis was authorized in 2016, with reimbursement implemented in September 2019, which may also have influenced subsequent patterns. *The 2024 figure is not final due to reporting delays.

Between 2021 and 2024, *Deja Tu Huella* has achieved significant milestones in improving HIV diagnosis and care across participating hospitals. Over a period of 4 years, 187 hospital EDs adopted the program, integrating HIV serology testing into routine practice. Of those, 114 hospital EDs incorporated the preconfigured electronic order sets and 56 the automated electronic alerts. This resulted in 223,659 HIV serologies requested and the identification of 2,357 new HIV diagnoses (Figure 5), with both variables showing a steady increase throughout the period (Table 1). Remarkably, by 2024 more than 1 in 5 new HIV diagnoses in Spain were made in EDs that implemented the *Deja Tu Huella* program, a landmark, program-driven achievement that underscores its national impact (Table 1, Figure 6). The positivity rate over these 4 years has averaged 1.15% with a decrease in 2024, probably due to the *Deja Tu Huella* impact decreasing the prevalence of occult infections. These efforts have also contributed to the prevention of HIV transmission, with a calculation of up to 9,428 avoided infections in 4 years (Figure 5) (24).

**Table 1:** Serologies, new diagnoses and positivity rate achieved by the *Deja Tu Huella* program from 2021 to 2024 (24).

Year	Serologies requested	New diagnoses	Positivity rate
2021	24,343	293	1.20%
2022	43,008	624	1.50%
2023	62,939	710	1.13%
2024	93,369	730	0.78%
Total	223,659	2,357	1.15% (average)

Beyond overall volumes, a descriptive, multicenter study that assessed all the serologies conducted in 10 participant hospitals of the *Deja Tu Huella* program (and the sister Catalonian program *Urgències VIHgila*) also revealed a consistent gender gap in ED HIV testing: men were tested more often than women despite representing a smaller share of ED attendances (0.69% vs. 0.47% testing probability). This pattern persisted across age groups and common syndromic triggers (CAP, MNS, and HZ), highlighting missed opportunities among women and pointing the way toward subsequent quality improvement actions (44).

.Anchored in this context, the clinical profile of patients diagnosed through ED screening adds further public health relevance. In a multicenter, retrospective evaluation across 17 Catalonian EDs, 23,105 serologies yielded 172 new diagnoses (positivity 0.7%); most individuals were assigned male at birth (88.4%; median age 39 years); sexual transmission was predominant (75.6%); and notably 57.8% of staged cases were acute infections while 24.2% presented with advanced HIV (45).

Similarly, a parallel analysis from the Madrid ED network reported 17,307 serologies and 169 new diagnoses (positivity 0.98%); the cohort included 72.2% migrants, 32.5% heterosexuals and 8.3% people who inject drugs, with late diagnosis in 48.6% and advanced disease in 27.6%. Despite 65.7% having at least one prior ED visit in the previous 5 years, only 30.8% had undergone HIV serology testing in that period (46).

As a corollary to these detection patterns, care cascade performance was robust across regions. Crucially, these diagnoses translated into rapid care engagement: in Catalonia, 82% of newly diagnosed patients were linked to specialist care and initiated antiretroviral therapy (ART) with a median of 9 days from the index ED visit (45), while in Madrid only 6.5% were not linked to care, with median times of 4 days to first HIV consultation and 8 days to ART initiation, mitigating the risks associated with late and advanced presentations (46).

These data taken together build on the strong linkage-to-care results and suggest that *Deja Tu Huella* has shifted ED practice from an opportunistic approach to standardized, high-yield screening, with order sets and automated alerts driving sustained growth in testing and earlier case finding; the observed sex differential highlights an equity gap to address. Overall, participating EDs are detecting more infections sooner and linking patients rapidly to ART. The program now offers a scalable template for ED-based HIV screening adaptable to other communicable disease priorities.

## Discussion

We are currently in a critical moment in our response to HIV infection. Countries that have approached or met the 95–95–95 targets are still challenged by a persistent reservoir of undiagnosed infections, and late diagnosis remains common (47). In this context, EDs offer a high-throughput, universal-access setting where indicator conditions cluster, making them a useful environment to convert missed opportunities into diagnoses (27, 48). Ending AIDS by 2030 requires earlier diagnosis, rapid linkage, and systematic testing in settings where undiagnosed prevalence renders screening cost-effective (41, 42, 47, 49).

Within this context, *Deja Tu Huella* translates consensus recommendations into operational practice through standardized clinical pathways, decision support, and education at scale. Delivery rests on the testing of indicator conditions—6 initially, later expanded to 9—implemented with informed opt-in consent, preconfigured test panels, indicator condition alerts, standardized result communication, defined linkage pathways, and continuous workforce training to minimize missed opportunities (24, 27, 33, 50).

Over its first 4 years, *Deja Tu Huella* supported more than 180 EDs, performed more than 223,000 serology tests, and diagnosed more than 2,350 new cases, suggesting that an opt-in, indicator condition strategy can achieve sustained, system-level impact when embedded in routine care (24). Moreover, *Deja Tu Huella* is playing a pivotal role in reversing the trend of new HIV diagnoses in Spain. Notably, the longstanding downward trajectory in reported cases, which had declined from a peak of 4,535 in 2014 to 2,980 in 2020, has been reversed. This decrease included a marked drop in 2020, coinciding with the nationwide introduction of pre-exposure prophylaxis (PrEP) in 2019. However, following the implementation of the *Deja Tu Huella* program, the trend has shifted (Figure 6). Hospitals participating in the initiative have reported a continuous annual increase in newly identified HIV-positive individuals (Table 1), contributing to a broader national rise in total diagnoses (Figure 6) (24). Importantly, this increase should not be interpreted as an actual rise in new infections, but rather as a reflection of the program’s effectiveness in identifying previously undiagnosed cases. By proactively targeting key populations and incorporating systematic testing into emergency care pathways, the *Deja Tu Huella* program is successfully revealing hidden infections. This represents a critical first step in the HIV continuum of care, enabling timely linkage to appropriate care and treatment services.

A relevant comparator is England’s national ED opt-out testing for blood-borne viruses in high-prevalence areas. This initiative, funded by the National Health Service (England) with £20 million over 3 years, normalizes a universal offer of testing that has delivered sizeable case findings, with additional yield for viral hepatitis. This model has been supported by dedicated central funding, robust IT automation, and national monitoring (20, 21). In practice, more than 1,000 new HIV diagnoses were recorded over the first 34 months of rollout (April 2022–January 2025) (51). Overall positivity rate was approximately 0.04%, and testing was performed in 57–67% of ED patients from whom blood samples were drawn; EDs accounted for around 14% of new HIV diagnoses nationally in 2022–2024 (52, 53). In contrast, Spain’s *Deja Tu Huella* emphasizes clinician engagement and indicator condition-based testing, requiring greater day-to-day professional involvement but fostering local ownership and adaptability (27, 33, 39, 50). This opt-in, targeted approach appears to achieve higher test positivity than England’s opt-out universal screening, with a positivity of approximately 1.26% in Spain in 2022–2023 and EDs contributing ~20% of all diagnoses (53). Both strategies have merits: crucially, the UK’s scale-up has been underpinned by ring-fenced central funding, whereas *Deja Tu Huella* has advanced with modest, distributed resources and unconditional support for tools and training, factors that shape the pace and scope of project implementation (20, 24).

The *Deja Tu Huella*-HIV network has also helped to better define HIV patient profiles and reveal structural gaps. EDs reach profiles that standard pathways miss. In Madrid, patients newly diagnosed in 17 *Deja Tu Huella* hospitals were more likely than national and regional aggregates to be migrants, heterosexuals, and women, and a substantial proportion presented late (46). In parallel, Catalonia’s multicenter ED program, also spanning 17 hospitals, reported that more than half of positive cases with available data were due to heterosexual transmission, approximately 58% were acute infections, and nearly 1 in 5 had advanced HIV (45). A study within the network also documented sex-based bias in HIV serology ordering, with fewer tests requested for people assigned female at birth (44).

Engagement, training, and awareness among ED physicians and nurses remain pivotal levers for increasing testing rates and adherence (40, 50). Stepwise implementation bundles that combine recurring education with automated EHR prompts and indicator condition alerts have been shown to produce substantial increases in HIV test ordering, particularly for STIs, MNS, CAP, and HZ (43). However, despite these gains, performance remains suboptimal for several indicator conditions such as MNS, CAP, and HZ, leaving a considerable opportunity for targeted interventions (34, 40, 43).

A distinctive strength of *Deja Tu Huella* is its sustainability. Coordinated national governance, regional leadership, routine reporting, pharmaceutical industry support (Gilead Sciences), and bidirectional dissemination (website, social media, newsletters) have resulted in uninterrupted operations with measurable outputs for over 4 years—an achievement without precedent in Spain’s ED setting and comparable only to the UK’s nationally backed program within a public health system. This partnership model is the basis for the infrastructure built on decision-support tools and educational content while preserving clinical independence (20, 24, 27, 33, 54).

Another defining feature of *Deja Tu Huella* is its reproducibility: a standardized yet flexible “change package” (indicator condition list, scripted consent, preconfigured orders/alerts, linkage pathways, and audit/feedback) that has been replicated across regions, hospital sizes, and heterogeneous EHRs with consistent implementation gains (50). Multi-site before/after evaluations and pragmatic audits show that the same bundle, consisting of education plus decision support and feedback, reliably increases testing adherence and case finding when transported to new sites (40, 50). External consistency across Catalonia and Madrid further supports generalizability of both yields and patient profiles, despite differences in local epidemiology and health service organization (46, 55). Network-level resources (templates, training curricula, dashboards) and mentorship from early adopters enable rapid onboarding and fidelity checks, reducing start-up variation and sustaining performance over time. Taken together, these elements indicate that *Deja Tu Huella* functions as a reproducible model, and not just a single-site intervention, with effects that persist after initial implementation (24).

Feasibility beyond EDs is high, and the next step could be scaling up to primary care and specialty outpatient clinics, leveraging the same change package with training, standardized pathways, and IT alerts. In addition, extending HIV testing with concurrent HCV serology, as already recommended in updated SEMES guidance, would amplify the public health yield and align with integrated blood-borne virus strategies (27, 33, 48, 50).

Future efforts, then, should consolidate 3 lines. First, scale-up with fidelity: continuous education, dashboards with site-level benchmarking, regular audits, and reinforced feedback loops (30, 50). Second, smarter targeting: piloting predictive-risk models (e.g., PREDICE) to prompt testing in high-yield presentations beyond the current indicator conditions, while preserving simplicity at the point of care (56, 57). Third, prevention linkage: formalize ED-to-PrEP referral pathways for patients with recent STIs, PEP users, or other high-risk profiles, making prevention the default strategy after a negative test (57, 58). These proposals are pragmatic extensions of the implementation of the *Deja Tu Huella* approach that, if executed in an equitable manner, should further accelerate progress toward the 2030 goals. The launch of a structured ED residency program in Spain in 2026, coinciding with the intake of a new cohort of residents, is expected to enhance the impact of the *Deja Tu Huella* program (59–61).

This review has several limitations. First, it is a narrative, non-systematic review. The external epidemiological and implementation studies used to provide broader context were selected pragmatically and were not identified through a formal systematic search, so some relevant publications may have been missed. Second, most quantitative estimates of program impact come from observational before–after or ecological analyses within the *Deja Tu Huella* network and from internal SEMES monitoring reports that have not undergone independent peer review. Also, participating hospitals joined the program on a voluntary basis and are likely to be early-adopting and more motivated centers, so they may not be fully representative of all Spanish EDs, which further limits generalizability. Together, these factors may limit causal inference and generalizability. Observational designs are inherently subject to selection bias, confounding and secular trends, and effect estimates should therefore be interpreted as associative rather than strictly causal. Potential conflicts of interest related to program funding and authors’ professional relationships are fully disclosed in the Funding and Conflict of Interest sections.

## Conclusion

The *Deja Tu Huella* program exemplifies a paradigm shift in HIV diagnosis by embedding targeted serological testing into routine clinical workflows, not as a screening tool, but as a high-efficiency differential diagnostic strategy. With a positivity rate of around 1%, it has been implemented in a large number of hospitals in Spain, supported by the novel use of automation to aid clinical decision-making in complex environments such as EDs.

This model not only prevents the omission of HIV serology when clinically indicated but also ensures that testing is performed where it is most likely to uncover an underlying cause. The strategy is aligned with national and regional health authority procedures and has achieved full consensus by stakeholders, including hospitals and health institutions.

The *Deja Tu Huella* program has proven to be an efficient and impactful system in Spain, accounting for over 20% of all new HIV diagnoses nationwide. Together, these features position *Deja Tu Huella* not only as a scalable and sustainable national initiative, but also as a potential model for other healthcare systems facing similar challenges, ultimately indicating the way forward in the fight to end HIV.

### The group member of SEMES Regional Coordinators

SEMES Regional Coordinators (Spanish Society of Emergency Medicine, SEMES, listed in the Supplementary materials).

## Footnotes

1
https://dejatuhuella.semes.org
