# Supplementary material for: Deja Tu Huella: a comprehensive review of a successful emergency department-based, HIV-targeted screening program in Spain and its impact on early diagnosis and public health
Source: Front Public Health. 2026 Jan 12;13:1734172. doi: 10.3389/fpubh.2025.1734172 (PMC12832552; doi:10.3389/fpubh.2025.1734172)
Supplement: Supplementary file 1 [file Table_1.docx]

**Supplementary Materials**

**Table S1.** Key publications resulting from the implementation of the *Deja Tu Huella* program.

| **Publication** | **Reference** |
| --- | --- |
| **[Recommendations for the early diagnosis of suspected**  **human immunodeficiency virus infection in the**  **emergency department and the referral of patients for**  **follow-up: a consensus statement of the Spanish Society**  **of Emergency Medicine (SEMES)]** | González Del Castillo J, et al. *Emergencias*. 2020;32:416-26. |
| [Screening for undiagnosed human immunodeficiency virus infection in Spanish emergency departments: current attitudes, inclination, and perception of obstacles related to the implementation of measures to improve detection] | Miró O, et al. *Emergencias*. 2021;33(4):254-64.  doi:[10.55633/s3me/044.2021](https://doi.org/10.55633/s3me/044.2021) |
| Map of sexually transmitted disease care in Spanish emergency departments | Miró O, et al. *Rev Esp Quimioter*. 2021;34(4):353-64.  doi:[10.37201/req/051.2021](https://doi.org/10.37201/req/051.2021) |
| [Efficiency of screening for human immunodeficiency virus infection in emergency departments: a systematic review and meta-analysis] | González Del Castillo J, et al. *Emergencias*. 2022;34(3):204-12.  doi:[10.55633/s3me/E069.2022](https://doi.org/10.55633/s3me/E069.2022) |
| [Targeted screening for human immunodeficiency virus infection in Spanish emergency departments: an analysis of epidemiologic and economic impact] | Moltó J, et al. *Emergencias*. 2022;34(1):21-28.  doi:[10.55633/s3me/E106.2022](https://doi.org/10.55633/s3me/e106.2022) |
| Evaluation of emergency department visits prior to an HIV diagnosis: Missed opportunities | Salmerón-Béliz OJ, et al. *Enferm Infecc Microbiol Clin (English ed)*. 2023;41(7):407-13.  doi:[10.1016/j.eimce.2021.12.010](https://doi.org/10.1016/j.eimce.2021.12.010) |
| [Emergency detection of HIV infection in patients consulting for conditions potentially related to occult infection: Initial results of the "Urgencies VIHgila" program] | Miró O, et al. *Rev Esp Quimioter*. 2023;36(2):169-79.  doi:[10.37201/req/085.2022](https://doi.org/10.37201/req/085.2022) |
| Targeted HIV testing in Spanish emergency departments | González Del Castillo J, et al. *The Lancet HIV*. 2023;10(9):e564.  doi:[10.1016/s2352-3018(23)00183-2](https://doi.org/10.1016/s2352-3018(23)00183-2) |
| Feasibility of a selective targeted strategy of HIV testing in emergency departments: a before-after study | González Del Castillo J, et al. *Eur J Emerg Med*. 2024;31(1):29-38.  doi:[10.1097/MEJ.0000000000001078](https://doi.org/10.1097/mej.0000000000001078) |
| [Impact of specialized training for emergency department nurses screening or undetected HIV infection: the “Urgències VIHgila” project experience] | Miró E, et al. *Emergencias*. 2024;36(3):188–96.  doi:[10.55633/s3me/019.2024](https://doi.org/10.55633/s3me/019.2024) |
| Analysis of the reasons for requesting HIV serology in the emergency department other than those defined in the targeted screening strategy of the "Urgencies VIHgila" program and its potential inclusion in a future consensus document | Miró O, et al. *Enferm Infecc Microbiol Clin* *(Engl Ed)*. 2024;42(9):492-500.  doi:[10.1016/j.eimce.2024.03.002](https://doi.org/10.1016/j.eimce.2024.03.002) |
| **[New recommendations of Spanish Society of Emergency Medicine (SEMES) for emergency department diagnosis of HIV infection based on results from Spain’s “Leave Your Mark” program]** | González Del Castillo J, et al. *Emergencias*. 2024;36(6):447-59. doi:[10.55633/s3me/094.2024](https://doi.org/10.55633/s3me/094.2024) |
| **Decalogue to promote the implementation and improvement of recommendations for the early diagnosis of HIV in Emergency Departments** | González Del Castillo J, et al*. Enferm Infecc Microbiol Clin (Engl Ed)*. 2024;42(5):267-71.  doi:[10.1016/j.eimce.2023.06.001](https://doi.org/10.1016/j.eimce.2023.06.001) |
| Role of emergency departments in HIV screening in Barcelona (Spain) and impact of a targeted opt-in strategy for HIV testing | Carbó M, et al. *Eur J Emerg Med*. 2025;32(4):278-87. doi:[10.1097/MEJ.0000000000001229](https://doi.org/10.1097/mej.0000000000001229) |
| [Hidden HIV diagnostic opportunity: a link to community-acquired pneumonia] | Ramio Lluch C, et al. *Emergencias*. 2025;37:74-5. |
| Characterization of new HIV diagnoses made in hospital emergency departments through the "Leave Your Mark" program | Calpe Delgado P, et al. *Enferm Infecc Microbiol Clin (Engl Ed)*. 2025;43(8):496-503.  doi:[10.1016/j.eimce.2025.05.006](https://doi.org/10.1016/j.eimce.2025.05.006) |
| Impact of three different interventions designed to improve  the implementation of an opt-in targeted strategy of HIV  testing in emergency departments | González Del Castillo J, et al. *HIV* Med. 2025;26(9):1429-44.  doi:[10.1111/hiv.70074](https://onlinelibrary.wiley.com/doi/10.1111/hiv.70074) |
| Sex differences in HIV testing in Catalan emergency departments | Robert N, Miró E, Llaneras J, et al. Sex differences in HIV testing in Catalan emergency departments. Eur J Emerg Med. Published online November 17, 2025. doi:10.1097/MEJ.0000000000001291 |
| [Emergency care and HIV diagnosis: an opportunity that health systems cannot afford to miss] | Miró O, et al. *Emergencias*. 2025;37:399-400. |
| Opt-in and opt-out strategies for HIV testing | Miró E, et al. *Lancet Reg. Health*. 2025;58:101482. |
| Characterization of new HIV diagnoses achieved in emergency departments using an opt-in strategy, Catalonia, Spain, July 2021 to March 2024 | Llaneras J, et al. *Eurosurveillance*. 2025. In press |
| **Total:** 21 original articles | |

Consensus documents in bold

***SEMES Regional Coordinators (Spanish Society of Emergency Medicine, SEMES)**

Raquel Rodríguez Calveiro, Servicio de Urgencias, Hospital Universitario Alvaro Cunqueiro, Vigo; Ana Morilla, Servicio de Urgencias, Hospital Universitario Central de Asturias, Oviedo; Francisco Mateos Chaparro, Servicio de Urgencias, Hospital Universitario Marqués de Valdecilla, Santander; Julio Javier Gamazo del Rio, Servicio de Urgencias, Hospital Universitario de Galdakao; Hugo Martínez Faya,  Servicio de Urgencias, Hospital Universitario de Navarra, Pamplona; Emili Gene, Servicio de Urgencias, Hospital Universitario Parc Tauli, Sabadell; Jesus Alvarez Manzanres, Servicio de Urgencias, Hospital Universitario Rio Hortega, Valladolid; Iria Miguens, Servicio de Urgencias, Hospital Clínico de Santiago de Compostela; Phillipe Calpe Delgado, Servicio de Urgencias, Hospital Universitario Puerta de Hierro, Madrid; Begoña Espinosa, Servicio de Urgencias, Corta Estancia y Hospitalización a Domicilio, Hospital General Dr. Balmis, Instituto de Investigación Sanitaria y Biomédica de Alicante (ISABIAL); Pedro Alarcon, Servicio de Urgencias, Hospital Universitario Reina Sofia, Murcia; Pascual Piñera, Servicio de Urgencias, Hospital Universitario Reina Sofia, Murcia; Nayra Cabrera, Servicio de Urgencias. Hospital Universitario Dr. Negrín, Gran Canaria; Lourdes Piedrafita, Servicio de Urgencias. Hospital de Manacor; Julian Lopez Alvarez,  Servicio de Urgencias del Hospital Universitario de Puerto Real, Cadiz; Federico Garcia, Servicio de Microbiología Clínica, Hospital Universitario Clínico San Cecilio, Granada, CIBERINFECC; Maria Jesus Perez Elias, Servicio de Enfermedades Infecciosas, Hospital Universitario Ramón y Cajal, IRYCIS, CIBERINFECC, Madrid; Santiago Moreno, Servicio de Enfermedades Infecciosas, Hospital Universitario Ramón y Cajal, IRYCIS, CIBERINFECC, Madrid; Reyes Velayos, Coordinadora Estatal de VIH/Sida de España; María Velasco, Servicio de Enfermedades Infecciosas, Hospital Universitario Fundación Alcorcón; Ricardo Juarez, Servicio de Urgencias, Hospital Universitario Nuestra Señora del Prado, Talavera de la Reina; Patricia Trenc, Servicio de Urgencias, Hospital Universitario Miguel Servet, Zaragoza.
